# Purification of Monoclonal Antibodies Using a Fiber Based Cation-Exchange Stationary Phase: Parameter Determination and Modeling

**DOI:** 10.3390/bioengineering3040024

**Published:** 2016-10-02

**Authors:** Jan Schwellenbach, Steffen Zobel, Florian Taft, Louis Villain, Jochen Strube

**Affiliations:** 1 Sartorius Stedim Biotech GmbH, Göttingen 37079, Germany; florian.taft@sartorius-stedim.com (F.T.); louis.villain@sartorius-stedim.com (L.V.); 2 Institute for Separation and Process Technology, Clausthal University of Technology, Clausthal-Zellerfeld 38678, Germany; zobel@itv.tu-clausthal.de (S.Z.); strube@itv.tu-clausthal.de (J.S.)

**Keywords:** monoclonal antibodies, downstream processing, cation-exchange chromatography, model, simulation, parameter determination

## Abstract

Monoclonal antibodies (mAb) currently dominate the market for protein therapeutics. Because chromatography unit operations are critical for the purification of therapeutic proteins, the process integration of novel chromatographic stationary phases, driven by the demand for more economic process schemes, is a field of ongoing research. Within this study it was demonstrated that the description and prediction of mAb purification on a novel fiber based cation-exchange stationary phase can be achieved using a physico-chemical model. All relevant mass-transport phenomena during a bind and elute chromatographic cycle, namely convection, axial dispersion, boundary layer mass-transfer, and the salt dependent binding behavior in the fiber bed were described. This work highlights the combination of model adaption, simulation, and experimental parameter determination through separate measurements, correlations, or geometric considerations, independent from the chromatographic cycle. The salt dependent binding behavior of a purified mAb was determined by the measurement of adsorption isotherms using batch adsorption experiments. Utilizing a combination of size exclusion and protein A chromatography as analytic techniques, this approach can be extended to a cell culture broth, describing the salt dependent binding behavior of multiple components. Model testing and validation was performed with experimental bind and elute cycles using purified mAb as well as a clarified cell culture broth. A comparison between model calculations and experimental data showed a good agreement. The influence of the model parameters is discussed in detail.

## 1. Introduction

Resin based packed bed chromatography is the most used unit operation in the downstream processing of protein therapeutics, due to its high protein binding capacity and separation efficiency [1,2,3,4]. Replacing resin based packed bed adsorbents with media relying on fast mass-transport, such as membrane adsorbers or monoliths, is often discussed as a promising alternative to improve the shortcomings of resin based media, namely high pressure drops and long residence times [5,6,7,8].

Recent advances in the production of fibers with shaped cross-sections based on extrusion processes offer the possibility to produce high-surface area fibers composed of different thermoplastic base materials [9]. As reported, a packed bed of these fiber materials, after a suitable surface modification, successfully addresses the shortcomings of hydrogel grafted non-porous adsorption supports regarding their often limited binding capacity, but maintain their advantages such as fast mass-transfer kinetics, high permeability, and accessibility of the binding sites for larger target molecules [10,11,12,13].

As demonstrated in numerous studies, the analysis and modeling of mass-transfer mechanisms and kinetic phenomena involved during chromatographic operations is an important tool regarding scale-up purposes [14], quality-by-design approaches [15,16], as well as process integration and optimization [17]. This approach has been proven to be effective for conventional resin based media [18,19,20,21,22] as well as for membrane adsorbers [23,24,25,26]. Numerous mathematical models have been proposed to describe the profiles obtained during chromatographic operations, characterized by different complexity levels in the description of the relevant mass-transport phenomena [27,28,29]. The general rate model (GRM) can be seen as the most universal model describing chromatography utilizing spherical porous particles. In this model, convection, axial dispersion, and all other relevant mass-transport resistances are taken into account [30]. This includes the external mass transfer of solute molecules from the mobile phase to the external surface of the adsorbent particle, the diffusion within the particle, and the adsorption-desorption process at the ligand sites, commonly described by various sorption isotherms like steric-mass-action (SMA) [16], Langmuir [31], or Freundlich [32]. The general rate model is often rearranged in simplified forms in which different mass transfer processes are expressed as a single term. One example is the lumped pore model (POR). It assumes that the adsorption-desorption process and the diffusion within the particle are very fast. This leads to the result that no radial concentration gradient within the particle is present. Simplifying only an average concentration value is assumed [33].

Within this study it is demonstrated that the POR model can be used to obtain a better understanding of the performance of the investigated novel stationary fiber-based phase under process conditions. Necessary model adaptions as well as the assignment, interpretation, and determination of model parameters are core themes of this study and are discussed in detail. All model parameters are determined by small scale experiments, correlations, or geometric considerations, independent from the chromatographic cycle, to increase the accuracy and predictive efficiency of the simulation results. For a first model validation and method establishment, purified monoclonal antibody (mAb) has been used as a model protein. This approach has been extended to a clarified mAb containing fermentation mixture. Model parameters for its main components (mAb-monomer, mAb-aggregates, and contaminants) could be determined via a combination of size exclusion and Protein A chromatography. The feasibility of this transfer of process design methods from chemical engineering to biotechnology suggests a huge benefit for process development and for the optimization of production processes.

## 2. Materials and Methods

### 2.1. Materials

#### 2.1.1. Chemicals

Hydrochloric acid (HCl, ACS reagent, 37%, Sigma Aldrich, St. Louis, MO, USA), sodium hydroxide (NaOH, >97%, Sigma Aldrich), sodium chloride (NaCl, >99.5%, Sigma Aldrich), acetic acid (AA, 100%, Roth, Karlsruhe, Germany), potassium dihydrogen phosphate (>99%, Roth), dipotassium hydrogen phosphate (>99%, Roth), pullulan (standard set, *M*_w_ 320–740,000 g/mol, Sigma Aldrich) were used in the following experiments. Ultrapure (UP) water was produced by an arium^®^pro ultra-pure water system (Sartorius Stedim Biotech GmbH, Goettingen, Germany). The monoclonal antibodies (mAb1 (MW: 145.5 kDa; isoelectric point (pI): 8.7) and mAb2 (MW: 144.2 kDa; pI: 8.3)) were kindly donated by Sartorius Stedim Biotech.

#### 2.1.2. Stationary Phase and Column Packing

**Figure 1:**
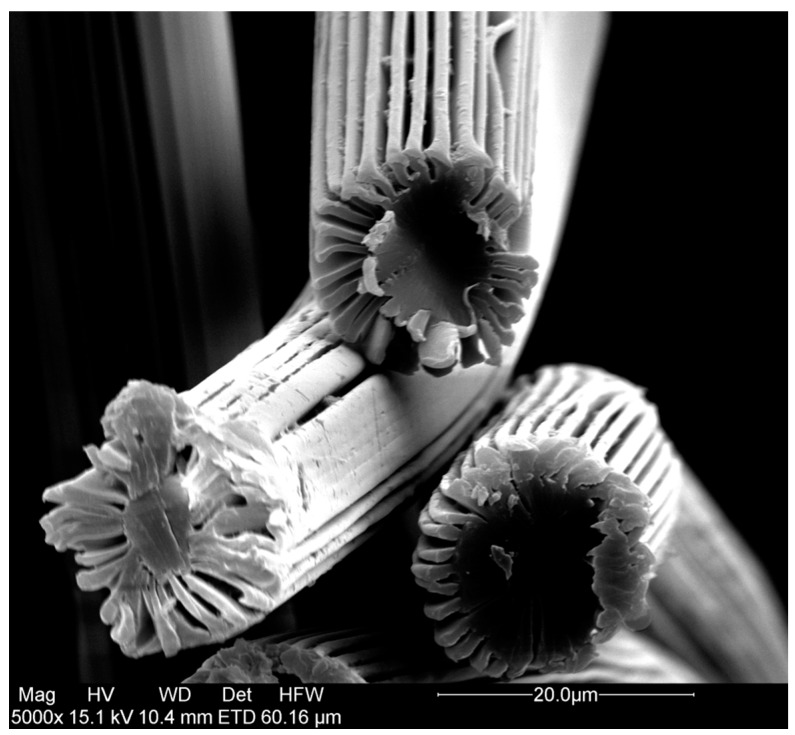
SEM photograph of a representative fiber sample.

Polyethylene terephthalate (PET) winged fibers (6 mm cut length, 3 denier per filament (dpf)) have been purchased from Allasso Industries. As determined by BET (Brunauer-Emmett-Teller) nitrogen adsorption experiments (Gemini V- Surface Area and Pore Size Analyzer, Micromeritics, Aachen, Germany) the fibers had a specific surface area of 2 m^2^/g. SEM photographs (Figure 1) revealed a diameter of about 15 µm and, as stated by the supplier, 32 winged-like projections extending radially from a main body region, forming channels with a width of about 500 nm.

The fibers were surface modified as reported previously [10]. Briefly, the fiber surface was modified by grafting glycidyl methacrylate (GMA) via surface-initiated atom transfer radical polymerization (SI-ATRP) and a subsequent derivatization leading to sulfonic acid groups. A ligand density of 235 ± 10 µmol/g was reached.

The preparation of a packed fiber bed was performed as described previously [10]. Briefly, in a representative packing procedure 0.825 g of PET-SO_3_^−^ fibers were, prior to column packing, slurried in ethanol, filtered off, and dried at 60 °C for 4 h to yield a fiber sample free of agglomerates. Afterwards, the fibers were transferred into a glass column (Goetec, Bickenbach, Germany, Superformance^®^ 150-10, 10 mm diameter), dried, and compressed to a bed height of 3 cm. This gave rise to a column volume of 2.36 mL and a fiber packing density of 0.35 g fiber material/mL column volume (CV). Afterwards, the resulting fiber bed was equilibrated with buffer at low linear flow velocities (50 cm/h, KPi-buffer 10 mM, pH = 7) before being characterized using an acetone tracer signal. Utilizing a packing procedure a height equivalent to a theoretical plate (HETP) value of 0.1 cm and asymmetry factor (AF) value of 1.8 could be achieved reproducibly.

### 2.2. Methods

All chromatographic experiments were carried out using a standard Agilent LC system (Agilent Technologies Inc., Santa Clara, CA, USA) compromised of a quaternary pump with degasser, tempered auto-sampler, a diode array (DA) detector, a conductivity detector, and a refractive index (RI) detector. A bed of fibers was packed into a glass column as described in Section 2.1.2 (packing density: 0.35 g/mL, bed height: 3 cm, column diameter: 1 cm) and connected to the standard Agilent LC system.

#### 2.2.1. Gradient Elution Experiments

The fiber bed was loaded with injections (100 μL) of a solution containing a purified mAb1 (4 mg/mL) or a clarified cell culture broth containing mAb2. Unbound components were washed out with the loading buffer A (KPi-buffer, 10 mM, 20 mM NaCl, pH = 6) in 5 CVs and the bound proteins were eluted and separated by passing elution buffer B (KPi-buffer, 10 mM, pH = 6, 500 mM NaCl) in a linear gradient of 0%–100% B in various CVs. The elution profile was recorded and analyzed by a diode-array detector (DAD) and conductivity detector. In the case of the clarified cell culture broth, the elution peak was fractionated (400 µL) and the eluted components were analyzed using Protein A and size exclusion chromatography (SEC).

#### 2.2.2. Inverse Size Exclusion Chromatography

Inverse size exclusion chromatography (iSEC) experiments were performed as previously conducted in numerous studies investigating various stationary phases [34,35,36]. Briefly, the fiber bed was equilibrated for 50 CVs of the desired buffer before being loaded with injections (100 µL) of a solution containing pullulan molecules (2 mg/mL) with a narrow molecular weight distribution. The mean molecular weight, directly linked to the mean hydrodynamic radius of the applied pullulan samples, was varied for every injection covering a wide range (*M*_n_ = 320–740,000 g/mol). The elution profile was recorded and analyzed by a RI detector.

#### 2.2.3. Isocratic Retention Experiments

The fiber bed was equilibrated for 50 CVs with buffer that had the desired NaCl concentration (KPi-buffer, 10 mM, pH = 6, 100–150 mM NaCl), before being loaded with injections (100 µL) of a solution containing purified mAb1 (4 mg/mL). The elution profile was recorded and analyzed by a DA detector.

#### 2.2.4. Batch Adsorption Experiments

Static batch adsorption experiments were performed as previously conducted in numerous studies [37,38,39]. Briefly, quantified samples, based on dry fiber mass, were immersed in a solution containing varying concentrations (0.0–5.0 mg/mL) of the solute molecule or the multi-component mixture. The concentration in the initial solution, the supernatant, and bound on the stationary phase was analyzed using Protein A chromatography and SEC.

#### 2.2.5. Size Exclusion Chromatography

Size exclusion chromatography was performed on a Yarra SEC-3000 (3 μm, 300 × 7.8 mm, Phenomex) SEC column. 5–100 µL of the investigated sample was injected at a flow rate of 1 mL/min using KPi-buffer (pH = 6.4, 100 mM Na_2_SO_4_) as the mobile phase buffer. The elution profile was recorded and analyzed by a DA detector. 

#### 2.2.6. Protein A Chromatography

Protein A chromatography was performed on a MAbPac column (Dionex). 5–100 µL of the sample was injected at a flow rate of 2 mL/min using PBS-buffer as binding and wash buffer. 10 mM HCl solution was used as elution buffer.

#### 2.2.7. Scanning Electron Microscopy

Scanning electron microscopy (SEM, FEI Quanta 200F, FEI, Waltham, MA, USA) was used to investigate the surface morphology of the membrane adsorbers. The fiber samples were coated with gold prior to investigation. The images were obtained using an acceleration voltage between 2–20 kV and a spot-size of 1.0–8.0 in a high vacuum. Secondary electrons were detected using an Everhart-Thornley Detector.

## 3. Theoretical

Modeling of hydrogel grafted chromatographic media with no internal porosity, especially membrane adsorbers, has been previously performed by several researchers [24,26,28]. In general, the chromatographic process is understood as a physical mechanism of mass-transport via convection and axial dispersion in the mobile phase. The mass transfer between the mobile phase and the stagnant phase surrounding the adsorbent is described by film diffusion [33]. Within the stagnant phase, a local equilibrium is assumed for each solute component between the binding sites and the surrounding fluid. Other transport mechanisms, such as axial diffusion or surface diffusion, are also present but it has already been demonstrated that they are not relevant in the case of membrane adsorbers and they are commonly disregarded [40]. Additionally, a suitable model for the entire chromatographic cycle must also account for the effects of fluid dynamics in the plant units. In the present work, this modeling approach was extended and adapted to describe a novel fiber based stationary phase.

### 3.1. Column Model and System Dispersion

**Figure 2:**
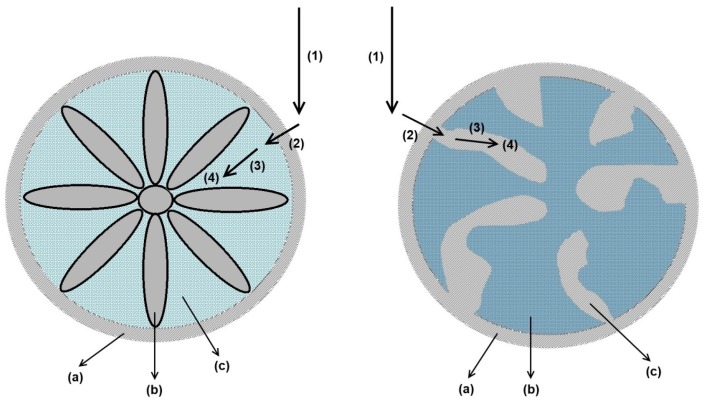
Comparision between mass-transport mechanisms for modified winged fibers (**left**); and porous resins (**right**). (1) Convective transport in mobile phase; (2) External film diffusion; (3) Hydrogel/Pore diffusion; (4) Adsorption. (a) External film; (b) Solid support matrix; (c) Hydrogel layer/Pore volume.

The structure of a packed bed of these fibers has been investigated and discussed in detail before. A bimodal structure within the fiber bed has been revealed, consisting of larger transport channels formed by the voidage between the fibers, and a hydrogel layer with porous properties [10]. Based on these findings, the fiber bed column is schematically considered as an ideal porous medium of total length *L* with uniform interstitial voidage $\varepsilon_{b}$ between the fibers and stationary phase distribution. The interstitial flow velocity *v* is constant and uniform over the fiber bed as a result of an effective flow distribution and collection at the column inlet and outlet. Therefore, velocity and concentration gradients in the radial direction are assumed to be absent. The fluid surrounding the grafted hydrogel layer, assumed to be constant and uniform throughout the column, is considered as the stagnant phase (Figure 2). The fraction of the hydrogel volume with respect to the total stationary phase volume, consisting of the hydrogel and fiber volume, is represented by the porosity of the stationary phase $\varepsilon_{p}$.

As estimated before, the thickness of the hydrogel layer using this surface modification technique reaches values >300 nm [10]. Based on this finding, it can be assumed that the channels formed by the winged-like extensions are completely filled with hydrogel bearing ligand sites. This leads to an analogy of the mass-transport phenomena between porous particles and the investigated fiber based medium (Figure 2).

Based on these findings, a transport-dispersive model (TDM) was chosen to describe the macroscopic mass transport through the fiber bed [41], accounting for convective mass transport of component *i* ($C_{i}\left(z,t\right))$ along the column with an interstitial velocity v and dispersion in space with an axial dispersion coefficient $D_{ax}$. The concentration exchange between the mobile and stagnant phase is described by an effective mass transfer coefficient $k_{eff}$, which lumps the contributions of the external film and internal diffusion processes, and the specific exchange area A. For the column inlet and outlet, Danckwerts boundary conditions have been applied, where $C_{i,D}\left(0,t\right)$ describes the injected concentration of component *i* at the column inlet at time *t*.

Following the approach of Morbidelli et al., the volume average concentration of component *i* within the stagnant phase $C_{f,i}\left(z,t\right)$ was described by a lumped pore procedure [33]. Its rate of change was determined by the exchange with the mobile phase and the adsorption equilibrium with the ligand sites.

The initial conditions were chosen as usual: $C_{i}\left(z,0\right)=\;C_{i}^{0}$ and $C_{f,i}\left(z,0\right)=\;C_{f,i}^{0}$. A summary of all relevant model equations can be found in Table 1.

The adsorption equilibrium between the stagnant fluid phase and the ligand sites was described through the multi-component Langmuir isotherm equation:

(5) $$q_{f,i}\left(z,t\right)=\frac{K_{eq,i}\cdot q_{max,i}\cdot C_{f,i}\left(z,t\right)}{1+\sum_{j=1}^{n}K_{eq,i}\cdot C_{f,j}\left(z,t\right)}$$

where $q_{f,i}$ denotes the volume average amount of component *i* bound on the stationary phase. The validity of this approach will be discussed in more detail in Section 4.1.2. In the case of only one component, Equation (5) reduces to a simple Langmuir expression. Following the work published by Yamamoto et al. and Forrer [42,43], the Langmuir parameters, namely the maximum binding capacity $q_{max,i}$ and the equilibrium binding constant $K_{eq,i}$, can be related to the salt concentration $c_{mod}$ within the fluid phase to describe the salt dependent binding behavior.

(6) $$q_{max,i}=a_{1}\cdot c_{mod}+a_{2}$$

(7) $$K_{eq,i}=b_{1}\cdot exp\left(-b_{2}\cdot c_{mod}\right)$$

In addition to the chromatographic medium, the experimental setup contains ancillary elements, such as valves, tubings, flow distributors, etc. As already demonstrated, the volume of these elements and the contribution to the flow non-idealities in the investigated system, at least at the laboratory or pilot plant scale, cannot be neglected [16,23]. A comparison between the simulation and experimental results in a quantitative way makes a description of the system dispersion effects necessary. As a standard approach was chosen to treat the system dispersion, the used model (Figure S1) as well as the parameter determination (Table S1) and model validation (Figure S2) can be found in the Supplementary Materials.

### 3.2. Parameter Determination

In the following section, the procedures that were used to determine the model parameters through independent measurement are discussed and presented. A major approach is represented by the conventional method of statistical moments. Applied to the chromatographic peaks resulting from a narrow rectangular pulse injection of the tracer into the system, this method is an effective approach to calculate the actual volume, voidage, and dispersion coefficient of the fiber bed or the external system.

For all signals, the first and second moments were measured and calculated as proposed by Haynes and Sarna [44] and corrected, if necessary, by subtracting the moments attributed to the extra-column volume of the HPLC system:

(8) $$\mu_{p,obs}=\frac{\int_{0}^{\infty }C_{d,i}\left(t\right)\cdot t\cdot dt}{\int_{0}^{\infty }C_{d,i}\left(t\right)\cdot dt}$$

(9) $$\sigma_{p,obs}^{2}=\frac{\int_{0}^{\infty }C_{d,i}\left(t\right)\cdot {\left(t-\mu_{p,obs}\right)}^{2}\cdot dt}{\int_{0}^{\infty }C_{d,i}\left(t\right)\cdot dt}$$

(10) $$\mu_{p}=\mu_{p,obs}-\mu_{HPLC}$$

(11) $$\sigma_{p}^{2}=\sigma_{p,obs}^{2}-\sigma_{HPLC}^{2}$$

where $\mu_{p}$ and $\sigma_{p}^{2}$ are the first and second moment of the tracer peak. $\mu_{p,obs}$ and $\sigma_{p,obs}^{2}$ are attributed to the whole system, whereas $\mu_{HPLC}$ and $\sigma_{HPLC}^{2}$ correspond only to the extra column volume. $C_{d,i}\left(t\right)$ represents the concentration of tracer *i* at the detector at time *t*.

#### 3.2.1. Axial Dispersion and Voidage of the Fiber Bed Column

Inverse size exclusion chromatography (iSEC) is a widely used method to determine the voidage and porosity of chromatographic media with respect to the molecule size [45]. It can also be used to calculate the pore size distribution using various models [35,46]. In this study, pullulan tracers with hydrodynamic radii *r*_H_ ranging from 0.5 to 20 nm were used to measure the accessible volume fraction of the cation-exchange fiber bed with respect to the tracer molecule size (Section 2.2.2). Following the moment analysis technique, a voidage value, depending on buffer conditions and molecule size, can be calculated using the following approach:

(12) $$\varepsilon =\frac{V}{F/\mu_{p}}$$

where ε represents the accessible volume fraction, *V* the column volume, and *F* the volumetric flow rate.

The axial dispersion coefficient is normally estimated through the moment analysis technique, and in the absence of direct measurements, can be calculated by empirical equations. However, the latter relationships are specifically developed for columns packed with spherical beads, and equivalent studies are not yet available for fiber beds.

Therefore, the axial dispersion coefficient has been measured using the moment analysis technique (Equations (8) and (9)) and can be calculated as follows:

(13) $$D_{ax}=\frac{\sigma_{p}^{2}\cdot v^{3}}{2L}$$

For uniform porous media completely filled with a single fluid phase, when the superficial velocity is uniform and the Peclet number > > 1, the axial dispersion coefficient reduces to

(14) $$D_{ax}=\alpha \cdot v$$

where α, commonly indicated as the dispersivity coefficient, can be understood as a geometrical parameter of the stationary phase, independent of the solute molecule. α can be determined by a measurement of $D_{ax}$ for different linear flow velocities using a linear regression.

#### 3.2.2. Effective Mass-Transfer Coefficient and Exchange Area

As stated above, the effective mass transfer coefficient $k_{eff}$ can be understood as a global mass-transfer coefficient, which can be described by an external and internal mass transfer coefficient.

(15) $$\frac{1}{\;k_{eff}}=\frac{1}{k_{ext}}+\frac{1}{k_{int}}$$

The external mass-transfer coefficient, characterizing the mass transport from the mobile phase to the stagnant phase surrounding the hydrogel layer, can be estimated by an evaluation of the Sherwood number *Sh*:

(16) $$Sh=\frac{k_{ext}\cdot d_{F}}{D_{m}}$$

where $d_{F}$ represents the fiber diameter and $D_{m}$ represents the molecular diffusion coefficient of the investigated solute molecule.

Using a correlation suggested by Wilson et al. [47], *Sh* can be calculated based on the Schmidt (*Sc*) and Reynolds (*Re)* number:

(17) $$Sh=\frac{1.09}{\varepsilon_{b}}\cdot Sc^{0.33}\cdot Re^{0.33}\;\mathrm{for}\;0.0015<Re<55$$

The internal mass-transfer coefficient $k_{int}$, characterizing the lumped mass transport within the stagnant phase surrounding the hydrogel layer, is commonly reduced to a time averaged value for spherical particles, especially the one proposed by Glueckauf [48]:

(18) $$k_{int}=5\frac{D_{eff}}{R_{p}}$$

where $D_{eff}$ denotes the effective molecular diffusion coefficient within the stagnant phase, which can be calculated as suggested by Kaczmarzski et al. and Guiochon et al. [18,49], and $R_{p}$ is the particle radius. This value has been interpreted as a mass-transfer resistance caused by a flat diffusive layer with a thickness of $R_{p}/5$ [50]. In this study, the diffusive layer thickness was characterized by the hydrogel layer filling the channels formed by the winged-like extensions of the fiber material. An estimation of $k_{int}$ can therefore be performed, if the hydrogel layer and the ligand distribution is assumed to be constant and uniform throughout the column. On average, the diffusive pathway that a solute molecule has to travel before reaching a ligand site within the hydrogel layer can be denoted as $r_{F}/2$. This leads to an expression for $k_{int}$:

(19) $$k_{int}=2\frac{D_{eff}}{r_{F}}$$

where $r_{F}$ denotes the fiber radius.

The determination of the effective exchange area *A* between the mobile and stagnant phase is often performed by a measurement of the specific surface area of the adsorbent. Due to hydrogel swelling and steric hindrance effects in the packed bed, this will most likely be an overestimation and represents the theoretical upper limit. A more realistic value for *A* can be estimated if the winged structure of the fiber material is assumed to be completely filled with the hydrogel layer. The effective exchange area is then better described by assuming a cylindrical fiber geometry with the radius $r_{F}$ (Figure 2):

(20) $$A=\frac{A_{fiber}}{V_{fiber}}=\frac{2\cdot \pi \cdot r_{f}\cdot h}{\pi \cdot r_{f}^{2}\cdot h}=\frac{2}{r_{f}}$$

#### 3.2.3. Salt-Dependent Binding Behavior

The adsorption equilibrium between the solute molecules in the stagnant phase and on the stationary phase was described by a Langmuir adsorption isotherm. The Langmuir parameters were obtained via static batch adsorption experiments. A Langmuir regression of the obtained data led to the required parameters for the target molecule. This procedure was repeated for at least five protein concentrations and four different salt concentrations $c_{mod}$ to obtain a parameter set describing the salt dependent binding behavior by regressing Equations (6) and (7).

The static batch adsorption approach was extended by a dynamic isotherm parameter determination approach for validation purposes. The salt dependency of the equilibrium binding constant for purified mAb was determined by isocratic pulse experiments varying the salt concentration in the buffer.

## 4. Results and Discussion

### 4.1. Parameter Determination

#### 4.1.1. Column Model

As presented before, a bimodal structure within the fiber bed was revealed, consisting of larger transport channels, formed by the voidage between the fibers, and a hydrogel layer with porous properties [10]. The characterization of the fiber bed, regarding its porous properties, was performed by applying inverse size exclusion chromatography as described in Section 2.2.2 and Section 3.2.1. Contributions of the extra column volume have been considered as shown in Equations (10) and (11).

**Figure 3:**
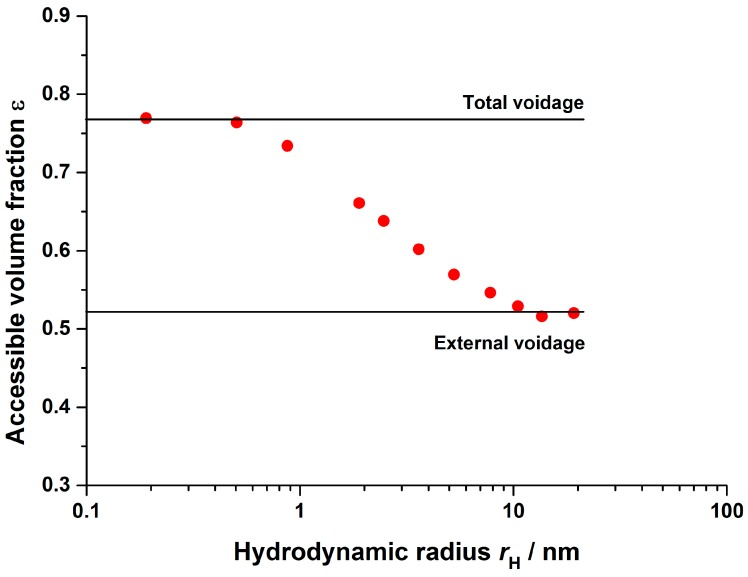
Accessible volume fractions depending on the pullulan tracer hydrodynamic radius. Buffer: 10 mM KPi, pH = 6, 20 mM NaCl.

As shown in Figure 3, small tracer molecules could completely access the fluid surrounding the hydrogel layer, reflecting the stagnant phase, as well as the volume in the larger transport channels, occupied by the mobile phase. The obtained boundary value for the accessible volume fraction therefore reflected the total voidage $\varepsilon_{T}$ of the fiber bed. Large tracer molecules were completely excluded from the hydrogel layer. The obtained boundary values for the accessible volume fraction reflected the external voidage $\varepsilon_{b}$, which was occupied by the mobile phase. Both boundary values were necessary to describe the porosity of the stationary phase $\varepsilon_{p}$:

(21) $$\varepsilon_{p}=\frac{\varepsilon_{T}-\varepsilon_{b}}{1-\varepsilon_{b}}$$

The results prove that in analogy to conventional bead based stationary phases [45], iSEC offers the possibility to determine and assign the bed voidage $\varepsilon_{b}$ and stationary phase porosity $\varepsilon_{p}$ for hydrogel grafted fiber based media in separate and independent measurements. This finding arises from the fact that the fiber bed structure shows properties comparable to porous beads, yet arising from different structural features (Figure 2).

**Figure 4:**
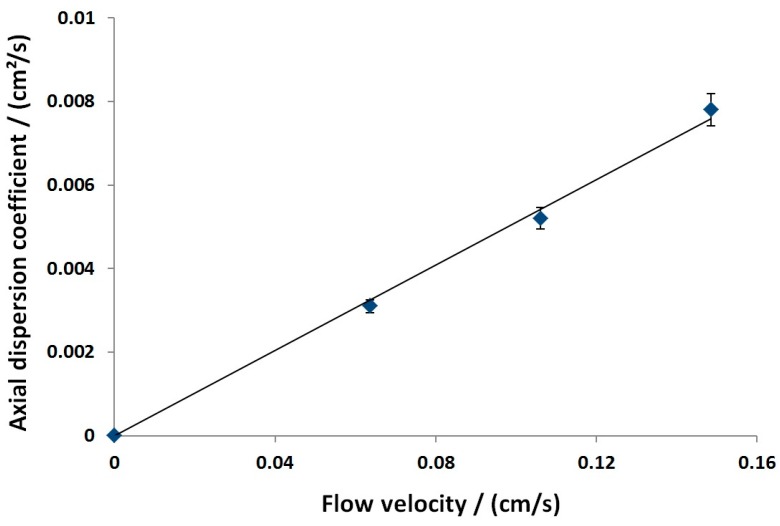
Dependency of the axial dispersion coefficient on the flow velocity. Buffer: 10 mM KPi, pH = 6, 20 mM NaCl.

As discussed in Section 3.2 and Section 3.2.1, the dispersivity coefficient α was determined using acetone tracer injections, while varying the flow rate/velocity. The method of moments was used for calculation. Contributions of the extra column volume were considered as mentioned above. In the investigated flow velocity range, the linear relation proposed in Equation (14) could be confirmed (Figure 4).

Shorter residence times within the column caused no major peak broadening and therefore indicated a relatively fast mass transport within the stationary phase. The slope reflected the dispersivity coefficient.

The effective exchange area and film mass-transfer coefficient, lumping the mass-transfer within the stationary phase, were determined following the procedure suggested in Section 3.2.2. Both parameters were combined resulting in the rate coefficient $k_{eff,A}$

(22) $$k_{eff,A}=\left(1-\varepsilon_{b}\right)\cdot A\cdot k_{eff}$$

The fiber material was assumed to be completely filled with the hydrogel layer (Figure 2). Therefore, the effective exchange area *A* per stationary phase volume was calculated based on the assumption of round cylindrical fiber geometry with the radius $r_{F}$ (Section 3.2.2). This value was independent of the investigated molecule.

However, the effective film mass-transfer coefficient $k_{eff}$ was dominated by the internal mass-transfer coefficient $k_{int}$. This led to values that strongly depended on the diffusion coefficient of the investigated molecule. It can be seen that the overall rate coefficient differed up to nearly two orders of magnitude depending on the molecule investigated.

A summary of all parameters regarding the fiber bed column, as well as the method used for determination, can be found in Table 2.

The peak shape of a simulated NaCl or acetone tracer signal was predominantly characterized by the axial dispersion coefficient. The determination via a moment analysis led to values that accurately described the measured peak shape.

**Figure 5:**
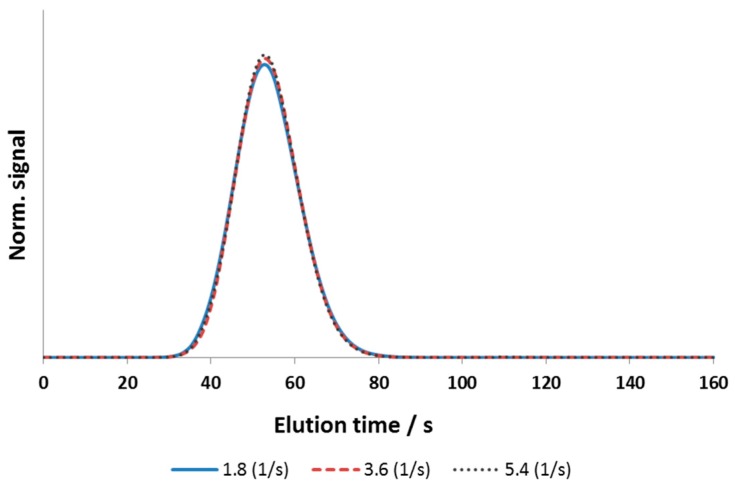
Influence of the rate coefficient *k*_eff,A_ on the peak shape of a simulated acetone tracer signal. Flow rate: 3 mL/min.

The rate coefficient $k_{eff,A}$, however, was rather high for acetone and NaCl. As expected, a variation of ±50% did not significantly influence the shape of the simulated peaks within the range of investigated linear flow velocities (Figure 5).

**Figure 6:**
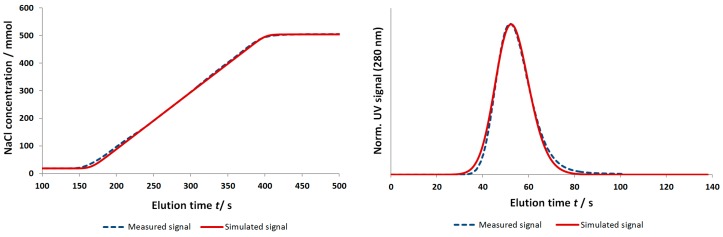
Comparison between measured and simulated tracer signal (right) and salt gradient (left). Tracer pulse: 100 µL acetone (2 vol %); flow rate: 3 mL/min; buffer: 10 mM KPi; pH = 6, 20 mM NaCl. Salt gradient: Buffer A: 10 mM KPi, pH = 6, 20 mM NaCl; Buffer B: 10 mM KPi, pH = 6, 500 mM NaCl; 5 CVs gradient duration; flow rate: 3 mL/min.

A comparison between a typical measured and simulated signal for an acetone tracer signal, as well as for a salt gradient, is shown in Figure 6. It can be demonstrated that the proposed column model, combined with the system dispersion, adequately described the fluid dynamic behavior. The centroid of elution was mainly determined by the voidage and porosity of the fiber bed and was independent of the investigated flow velocity. The conformity between the simulated and measured signals regarding the centroid of elution validated the iSEC approach to determine the assigned porosity parameters of the fiber bed. The high accuracy of the simulation results in general rendered the assignment, interpretation, and independent determination of parameters via separate experiments or correlations as a viable approach for a complete characterization of the investigated fiber based system. A validated description of the fluid dynamic behavior of the whole system, including the system dispersion, could be obtained, which enabled an independent detailed investigation of the salt depending binding for different target molecules.

#### 4.1.2. Salt-Dependent Binding Behavior

The charged hydrogel layer, grafted on the fiber surface, formed a three-dimensional network. The adsorption of target molecules was generally determined by the charge interaction but was also limited by the available volume within the hydrogel. The description of the binding behavior was commonly performed using an adsorption isotherm model. Many different examples can be found in the literature, such as the steric mass action or the Langmuir isotherm [26,51]. For hydrogel grafted ion-exchange media, the Langmuir isotherm model has been proven to sufficiently describe the observed adsorption behavior. This might be contradictive, as the Langmuir isotherm describes in theory the adsorption of a monolayer on an energetically homogenous surface [52]. The commonly observed multi-layer adsorption within a hydrogel does not correspond well with the assumptions of the Langmuir model. Nevertheless, an analogy between the energetically homogenous surface and the homogenous binding volume within the hydrogel layer could be proposed. The maximum binding capacity was reached when the available binding volume within the hydrogel was filled, just as a full surface coverage determines the maximum binding capacity in the Langmuir model. This analogy might be the reason for the good agreement of the measured salt-dependent adsorption behavior and the Langmuir regression.

**Figure 7:**
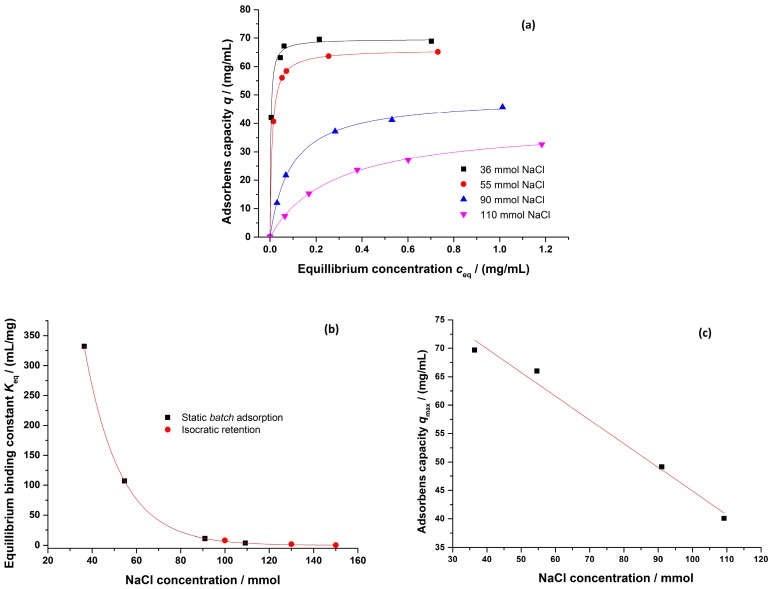
(**a**) Binding isotherms determined for purified mAb1; (**b**) Exponential regression of the equilibrium binding constant dependence on the NaCl concentration; (**c**) Linear regression of the maximum adsorbens capacity dependence on the NaCl concentration. Buffer: 10 mM KPi, pH = 6, various NaCl concentrations.

As described in Section 2.2.4, the adsorption data for a purified mAb (mAb1) was determined at four different NaCl concentrations using Protein A chromatography, and was calibrated using an IgG standard solution. As seen in Figure 7, the description of the measured adsorption data with a Langmuir regression was very good (*R*^2^ ≥ 0.96). The resulting Langmuir parameter sets were regressed as suggested by Yamamoto et al. and Forrer [42,43] to describe their dependency on the salt concentration. The equilibrium binding constant was also determined by applying isocratic retention experiments. Because of its strong dependence on the salt concentration, no experimental data was available at salt concentrations <100 mM due to major peak broadening. The conformity of the parameter sets measured by a static and a dynamic method supports the validity of both approaches.

**Figure 8:**
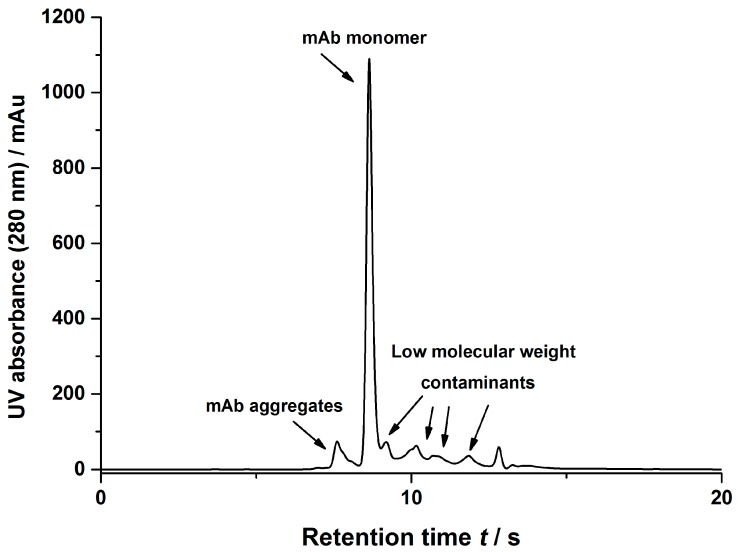
Representative size exclusion chromatogram of the clarified cell culture broth including an assignment of the different species present.

This procedure was transferred to a clarified cell culture broth containing mAb2. A representative size exclusion chromatogram of said solution is shown in Figure 8. As often discussed, the clarified cell culture broth contained, besides the mAb monomer and its aggregates, a high number of different contaminants, such as mAb fragments, host cell proteins (HCPs), and DNA fragments. To obtain the salt dependent adsorption data, the components of the cell culture broth were condensed to three main classes, namely mAb monomer, mAb aggregates, and low molecular weight contaminants (Figure 8). As described in Section 2.2.4, batch adsorption measurements were conducted and analyzed using a combination of analytic techniques. Protein A chromatography yielded data for the complete amount of mAb, including monomers and aggregates. SEC allowed for differentiation between the mAb monomers and aggregates and yielded data for the condensed low molecular weight contaminants spectrum. Both methods were calibrated using an IgG standard. As no direct calibration was available for the whole spectrum of low molecular weight contaminants, the IgG calibration was applied. Therefore, the measured concentrations have to be understood as pseudo-concentrations and not as absolute values.

**Figure 9:**
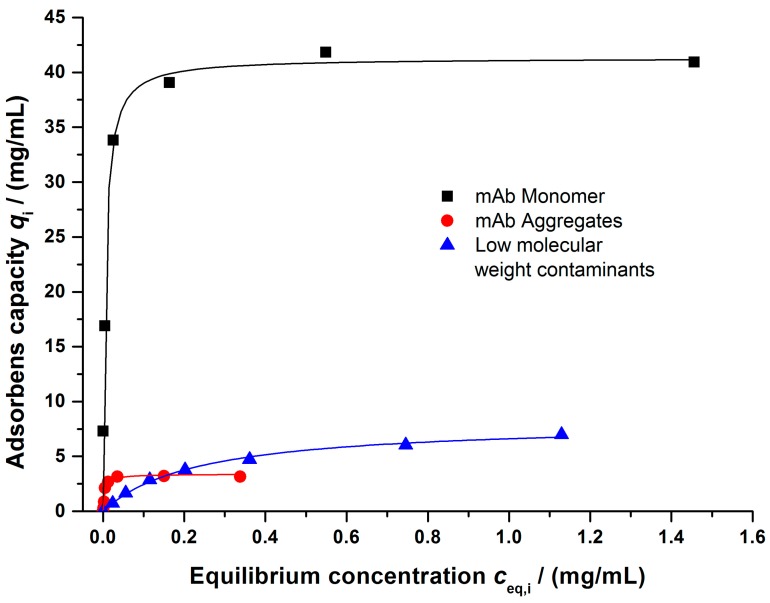
Binding isotherms determined for the main components of the clarified cell culture broth: mAb momomer, mAb aggregates, and low molecular weight components. Buffer: 10 mM KPi, pH = 6, 40 mM NaCl.

As observed for the purified mAb model system, the obtained salt dependent adsorption data for all main components can be described by a Langmuir regression (Figure 9). Furthermore, a comparison between the data obtained by Protein A chromatography and SEC showed a good agreement for the total amount of mAb. Additionally, the salt dependencies of the Langmuir parameters followed the relations suggested by Yamamoto et al. and Forrer et al. These results supported a successful binding parameter determination, even for a complex multi-component mixture.

### 4.2. Model Validation

#### 4.2.1. Gradient Elution Experiments

For model validation, the fluid dynamic and binding parameters, determined by small scale experiments independent from the chromatographic cycle, were used to predict the gradient elution behavior of purified mAb1 as well as for the clarified cell culture broth, containing mAb2. As described in Section 2.2.1, the fiber bed was loaded with injections of the investigated solution, before a salt gradient of varying steepness was applied using different flow rates (3–7 mL/min) to elute the bound molecules.

**Figure 10:**
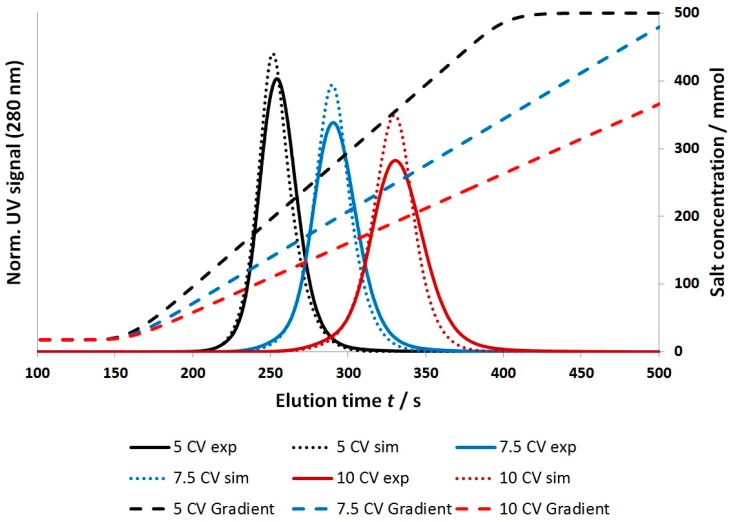
Comparision between the measured and simulated signals for a bind and elute experiment using purified mAb1. Binding and wash buffer: 10 mM KPi, pH = 6, 20 mM NaCl; elution buffer: 10mM KPi, pH = 6, 500 mM NaCl; 5–10 CVs gradient duration; flow rate: 3 mL/min.

Figure 10 shows that the comparison of the simulation and experimental data led to a sufficient result for purified mAb1 (*R*^2^ ≥ 90) at a flow rate of 3 mL/min.

The proposed model correctly described the centroid of elution, the total peak area, and the peak broadening with decreasing gradient steepness. This observation was also true for higher flow rates up to 7 mL/min. Furthermore, at higher flow rates an additional minor peak broadening of the experimental signals was observed, which was also predicted by the simulation.

**Figure 11:**
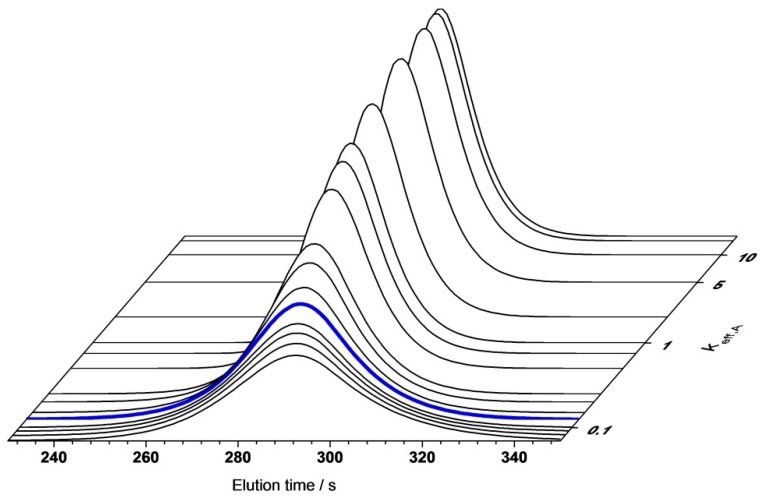
Sensitivity analysis of rate coefficient *k*_eff,A_. 7.5 CVs gradient elution; flow rate 3 mL/min. The blue line represents a rate coefficient of 0.129 (1/s).

A systematic deviation can be found as the predicted elution profiles tend to be slightly too narrow compared to the experimental results. A sensitivity analysis of the model parameters led to the conclusion that the peak broadening was significantly influenced by $D_{ax}$, the salt-dependency of the Langmuir parameters, and $k_{eff,A}$. As the Langmuir parameters were determined by direct measurements, including a static and dynamic method, and $D_{ax}$ was validated by tracer experiments under non-binding conditions (Section 4.1), the observed deviation can most likely be attributed to an overestimation of the rate coefficient $k_{eff,A}$. A sensitivity analysis of $k_{eff,A}$ was performed and is shown in Figure 11. The investigated range covered values ranging from 15.5 to 0.065 (1/s). The highest value chosen was based on a calculation assuming that the effective exchange area was given by the specific surface area of the winged fibers as determined by BET measurements and that the diffusive layer thickness equaled the hydrogel layer thickness (340 nm) as calculated previously [10]. The lowest value was calculated based on the assumptions in Section 3.2.2, but was reduced by 50% to account for additional effects such as an overestimation regarding the effective molecular diffusion coefficient and improper assumptions with respect to the diffusive layer thickness and the effective exchange area. The blue line represents a $k_{eff,A}$ value of 0.129 (1/s) as used for the simulation results shown in Figure 10.

It can be seen that the rate coefficient was a very sensitive parameter for values <1 (1/s). Higher values described a very fast mass transfer rate which rendered the influence of $k_{eff,A}$ insensitive in the range of the investigated flow velocities.

The optimal value for $k_{eff,A}$ was estimated by simulation to be 0.105 (1/s). This equaled a deviation of −20% compared to the calculated value. These results, as shown in Figure 10 and Figure 11, suggest that the determination of $k_{eff,A}$ based on geometrical considerations and correlations was a viable approach.

Another factor which can additionally influence the peak width is the presence of mAb charge variants. As the binding parameters, determined by static batch adsorption, only described an average for all components that were present in the solution, if they were not separately analyzed, the elution peaks for the different components would be predicted as a single averaged peak. Despite these small deviations, the a priori prediction of the elution behavior of a purified mAb could be achieved with high accuracy.

**Figure 12:**
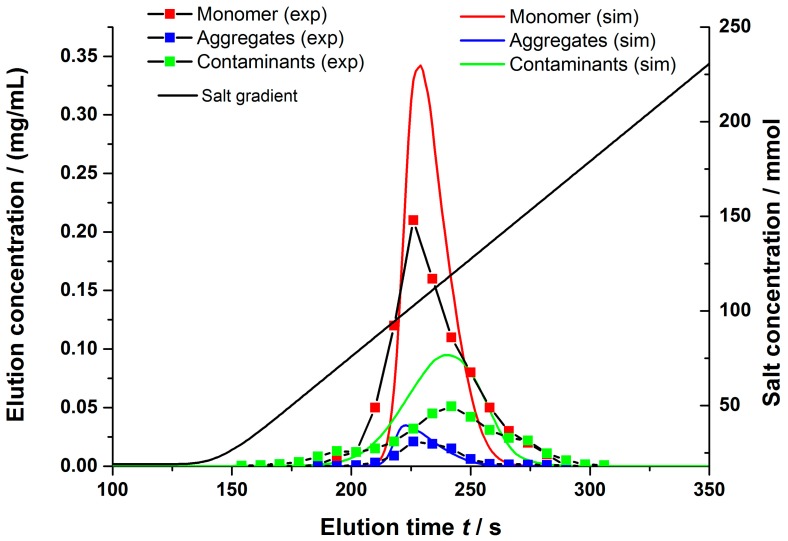
Comparision between the measured and simulated signals reagarding the main components for a bind and elute experiment using clarified cell culture broth. Binding and wash buffer: 10 mM KPi, pH = 6, 20 mM NaCl; elution buffer: 10 mM KPi, pH = 6, 500 mM NaCl; 10 CVs gradient duration; flow rate: 3 mL/min.

The approach of a parameter determination based on small scale experiments, combined with geometrical considerations and correlations regarding the stationary phase, was proven to be feasible. This approach was extended to a clarified cell culture broth. As demonstrated in Section 4.1.2, the determination of binding parameters for the different main components could be achieved by a suitable combination of analytic techniques. A multi-component Langmuir isotherm was used to describe the competitive binding on the stationary phase. This allowed for an a priori prediction of the separation performance of the investigated novel stationary phase under process conditions. The elution was fractionated and analyzed regarding its content of the main components. A representative result is shown in Figure 12. It can be seen that the proposed model again correctly described the centroid of elution for all investigated components. The simulation predicted no major separation between the mAb monomer and aggregates, as it had been validated by measurements. A nearly constant ratio of monomers and aggregates could be found in the elution fractions. As predicted by the simulation, the majority of the low molecular weight components, however, showed an elution at higher salt concentration. Compared to the simulation results, the measurements showed no single elution peak but a more complex elution behavior. An explanation for this observation can be found in the lumping procedure of the low molecular weight components, by treating them as one component. Nevertheless, the centroid of elution was predicted with a high accuracy and offers an insight in the separation performance of this novel stationary phase even under process conditions. Similar results could be found if the gradient steepness was varied (5 and 7.5 CV).

## 5. Conclusions

Within this work, the lumped pore model has been studied and adjusted to predict the purification performance of a novel fiber based chromatographic cation-exchange medium. The suggested model can describe all stages of a chromatographic cycle, namely adsorption, washing and elution, as well as the external system dispersion.

The physico-chemical base of the applied model equations allowed for the assignment of a physical meaning to every parameter. This enabled a determination independent from the chromatographic cycle in small scale experiments to increase the accuracy and predictive efficiency of the simulation results.

It was demonstrated that this procedure led to an adequate description of the fluid dynamic behavior of a packed fiber bed at various volumetric flow rates. Furthermore, the gradient elution of a purified mAb could be predicted a priori by applying different gradients, while the salt dependent binding parameters could be gathered from static batch adsorption measurements. This approach has been proven to be feasible to obtain the binding parameters for a complex multi-component mixture.

The application of the model to describe the separation performance of the novel stationary phase by using clarified cell culture broth yielded a good agreement between the simulation and the measurements. Despite the fully predictive nature of the model, the separation performance can be described adequately without the need for additional fitting parameters. The proposed model as well as the parameter determination can therefore be seen as validated for the investigated systems. This transfer of process design methods from chemical engineering to biotechnology can provide a significant benefit for optimization and scale-up procedures.

## Figures and Tables

**Table 1:** Summary of the relevant model equations.

Model Equations	Description
$\frac{\partial C_{i}\left(z,t\right)}{\partial t}=-v\frac{\partial C_{i}\left(z,t\right)}{\partial z}+D_{ax}\frac{\partial^{2}C_{i}\left(z,t\right)}{\partial z^{2}}$ $-\frac{\left(1-\varepsilon_{b}\right)}{\varepsilon_{b}}\cdot A\cdot \;k_{eff}\;\cdot \left(C_{i}\left(z,t\right)-C_{f,i}\left(z,t\right)\right)$	(1) Mass transport in the mobile phase
$\varepsilon_{p}\frac{\partial C_{f,i}\left(z,t\right)}{\partial t}+\left(1-\varepsilon_{p}\right)\frac{\partial q_{f,i}\left(z,t\right)}{\partial t}=A\cdot \;k_{eff}\;\cdot \left(C_{i}\left(z,t\right)-C_{f,i}\left(z,t\right)\right)$	(2) Mass transport in the stagnant phase
$v\cdot C_{i}\left(0,t\right)-\;D_{ax}\frac{\partial C_{i}\left(0,t\right)}{\partial z}=v\cdot C_{i,D}\left(0,t\right);t>0$	(3) Boundary condition—column inlet
$\frac{\partial C_{i}\left(L,t\right)}{\partial z}=0;\;t>0$	(4) Boundary condition—column outlet

**Table 2:** Summary of the parameters determined for the column characteristics and mass transport mechanisms.

Parameter	Value		Method of Determination
$\varepsilon_{T}$	0.76		Inverse size exclusion chromatography
$\varepsilon_{b}$	0.54		
$\varepsilon_{p}$	0.48		
α/cm	0.051		Moment analysis
	Molecular Diffusion Coefficient/(m^2^/s)	$k_{eff,A}/$(1/s)	Correlation/Geometrical consideration (Section 3.2.2)
NaCl	1.99 × 10^−9^	6.44	
Acetone	1.14 × 10^−9^	3.67	
mAb	4.00 × 10^−11^	0.129	

## Supplementary Materials

The following are available online at http://www.mdpi.com/2306-5354/3/4/24/s1. Purification of monoclonal antibodies using a fiber based cation-exchange stationary phase: parameter determination and modeling. Figure S1: experimental setup schematization displaying the modeling approaches, Figure S2: comparison between a measured and simulated tracer signal for the external system dispersion. Tracer pulse: 100 µL acetone (2%), flow rate: 3 mL/min, buffer: 10mM KPi, pH = 6, Table S1: summary of the parameters determined for the external system dispersion using different volumetric flow rates.
