# Supplementary material for: Purification of Monoclonal Antibodies Using a Fiber Based Cation-Exchange Stationary Phase: Parameter Determination and Modeling
Source: Bioengineering (Basel). 2016 Oct 2;3(4):24. doi: 10.3390/bioengineering3040024 (PMC5597267; doi:10.3390/bioengineering3040024)
Supplement: Supplementary file 1 [file bioengineering-03-00024-s001.pdf]

bioengineering

Supplementary Materials: Purification of Monoclonal Antibodies Using a Fiber Based Cation-Exchange Stationary Phase: Parameter Determination and Modeling

Jan Schwellenbach, Steffen Zobel, Florian Taft, Louis Villain and Jochen Strube

System Dispersion: Parameter Determination and Model Validation

As commonly discussed, a simple model to simulate the system influence is a series of a stirred tank (ST) and a distributed plug flow pipe (DPF), with the volumes V_{ST} and V_{DPF} [1,2]. The total external system volume V_{SYS} can therefore be summarized as:

$$V_{SYS} = V_{ST} + V_{DPF} \tag{S1}$$

As shown in Figure S1 the contribution of the external system is condensed in front of the column, meaning that the exit of the system dispersion model equals the inlet of the column model. The mass balance equations for both systems are given as usual:

$$\frac{\partial C_{i,out}^{ST}}{\partial t} = \frac{F}{V_{ST}}\left(C_{i,in}^{ST} - C_{i,out}^{ST}\right) \tag{S2}$$

$$\frac{\partial C_i^{DPF}(z,t)}{\partial t} = -v\frac{\partial C_i^{DPF}(z,t)}{\partial z} + D_{ax,DPF}\frac{\partial^2 C_i^{DPF}(z,t)}{\partial z^2} \tag{S3}$$

where F denotes the volumetric flowrate, $C_{i,out}^{ST}$ and $C_{i,in}^{ST}$ the outlet and inlet concentration of component i in the ST, $C_i^{DPF}(z,t)$ is the concentration of component i in the DPF, and $D_{ax,DPF}$ is the axial dispersion coefficient of the DPF.

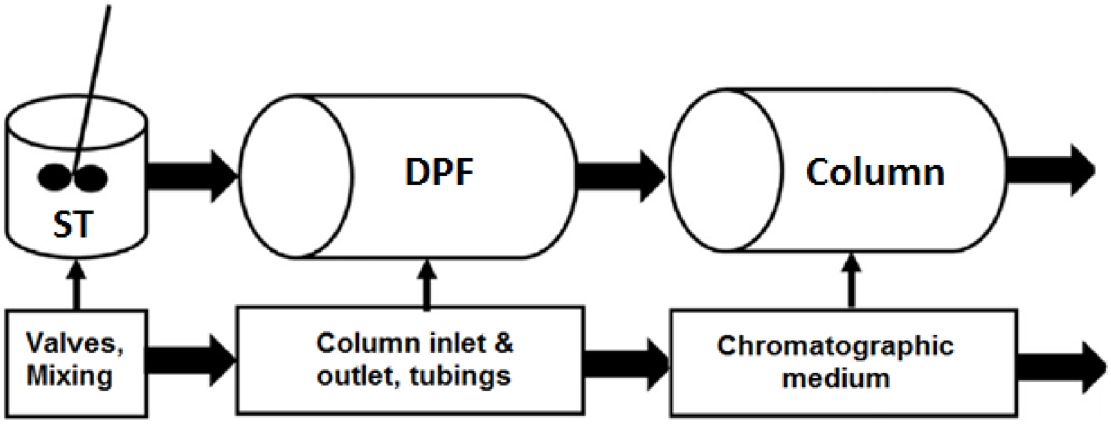

Figure S1. Experimental setup schematization displaying the modeling approaches.

The system dispersion model contains four unknown parameters that have to be determined, namely V_{SYS}, V_{ST} , V_{PFP}, and $D_{ax,DPF}$. The total volume of the external system V_{SYS} can be determined in the absence of the fiber bed column by applying an analysis of the first moment of a tracer signal. As suggested by Boi et al. [2], a regression applying Equation (S2) and (S3) to the concentration profile of a tracer substance leaving the system after a pulse injection, leads to the missing parameters to describe the system dispersion model.

To determine values for the external system dispersion model, pulse injections of acetone (2 vol % in RO-water) or mAb (4 mg/mL in KPi-buffer, 10 mM, 20 mM NaCl, pH = 6) were carried out in the absence of the fiber column. The experiments were performed using different volumetric

flow rates and buffer conditions. The resulting peak signals were evaluated following the method of moments and regressed to obtain the necessary values. As already demonstrated by Boi et al. a slight dependence of V_{DPF} and $D_{ax,DPF}$ on the volumetric flow rate were found, which might be attributed to a time delay associated to valve switching (Table S1) [2]. However, no dependence on the buffer conditions or the tracer molecule could be found. A comparison between a typical measured and simulated tracer signal is shown in Figure S2, displaying the ability of the external dispersion model to describe the observed fluid dynamic behavior. Identical results were found at higher flow rates. The high accuracy of the simulation results ($R^2 = 0.95$) proves the validity of the approach to combine a ST and DPF to fully characterize the complex external system. The necessary parameters could be obtained independent of the chromatographic cycle with low experimental effort.

This led to a validated description of the external system dispersion, which is mandatory for a correct and decoupled consideration of the fiber bed column.

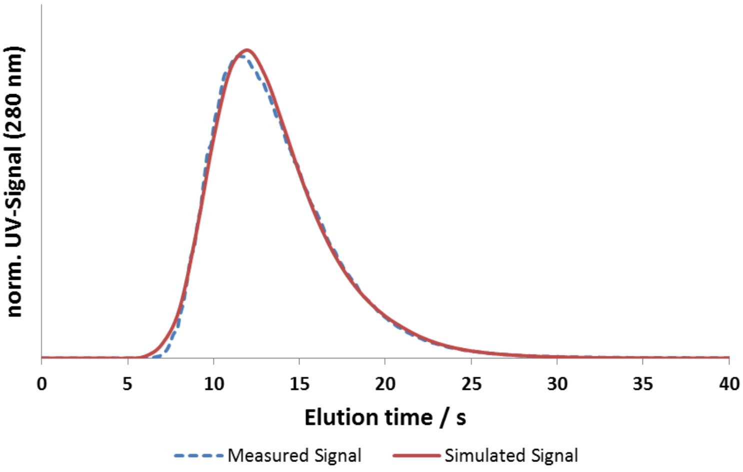

Figure S2. Comparison between a measured and simulated tracer signal for the external system dispersion. Tracer pulse: 100 µL acetone (2%), flow rate: 3 mL/min, buffer: 10mM KPi, pH = 6.

Table S1. Summary of the parameters determined for the external system dispersion using different volumetric flow rates.

Volumetric Flow Rate/mL/min	V_{SYS}/mL	V_{ST}/mL	V_{DPF}/mL	$D_{ax,DPF}$/(cm²/s)
3	0.60	0.14	0.46	1.1
5	0.61	0.14	0.47	1.05
7	0.63	0.14	0.49	1.06

References

1. Shekhawat, L.K.; Manvar, A.P.; Rathore, A.S. Enablers for QbD implementation: Mechanistic modeling for ion-exchange membrane chromatography. *J. Membr. Sci.* **2016**, *500*, 86–98.
2. Dimartino, S.; Boi, C.; Sarti, G.C. A validated model for the simulation of protein purification through affinity membrane chromatography. *J. Chromatogr. A* **2011**, *1218*, 1677–1690.

© 2016 by the authors. Submitted for possible open access publication under the terms and conditions of the Creative Commons Attribution (CC-BY) license (http://creativecommons.org/licenses/by/4.0/).
